# Case Report: Extensive Phosphorylation of Interleukin-1 Receptor-Associated Kinase 4 in a Patient With Schnitzler Syndrome

**DOI:** 10.3389/fimmu.2020.576200

**Published:** 2020-09-30

**Authors:** Isabel Hodl, Philipp Bosch, Barbara Dreo, Martin H. Stradner

**Affiliations:** Division of Rheumatology and Immunology, Department of Internal Medicine, Medical University of Graz, Graz, Austria

**Keywords:** Schnitzler syndrome, autoinflammatory disease, interleukin-1 receptor associated kinase 4, flow cytometry, interleukin 1, Castleman disease

## Abstract

Schnitzler syndrome (SchS) is a rare autoinflammatory disease, characterized by urticarial rash, recurrent fever, osteo-articular pain/arthritis with bone condensation, and monoclonal gammopathy. Diagnosis may be difficult due to overlapping signs with other diseases. Here, we describe the case of a 62-year-old man with SchS, who was initially misdiagnosed with multicentric Castleman disease (MCD). As excessive release of IL-6 is characteristic of MCD, in contrast to IL-1 in SchS, we measured the phosphorylation of intracellular signaling proteins of the respective pathways by flow cytometry. We found a distinct increase of phosphorylated IRAK-4 in our patient’s B cells and monocytes while phosphorylation of STAT-3 was low, suggesting predominant IL-1 signaling. In accordance with these results and the classification criteria, we established the diagnosis of SchS instead of MCD and commenced therapy with the IL-1 receptor antagonist anakinra. We observed a rapid remission of signs accompanied by a reduction of phosphorylated IRAK-4 to normal levels. In conclusion, we propose phosphorylated IRAK-4 in B cells and monocytes as a potential marker for diagnosis of SchS and for treatment response to IL-1 blockade.

## Introduction

Schnitzler syndrome (SchS) is a rare autoinflammatory syndrome, first described in 1972 by Liliane Schnitzler with currently about 300 cases reported worldwide (1). Clinically, it is characterized by urticarial rash, fever, swollen lymph nodes, bone and/or joint pain. In addition, elevated C-reactive protein (CRP), leukocytosis, and monoclonal gammopathy of immunoglobulin M (IgM) or IgG type are hallmarks of SchS (2). These signs and symptoms can be utilized to classify SchS (3).

The pathophysiology of SchS is elusive. Previously, it has been hypothesized that immunoglobulins produced by expanded B cell clones may cause the signs of SchS (2). Monoclonal immunoglobulins are found at the dermoepidermal junction and in capillary walls, which may lead to chronic urticaria (4). Furthermore, excessive production of interleukin 1 (IL-1) is found in SchS and therapy based on an IL-1 blockade leads to remission of symptoms within hours (5). It remains unclear if this imbalance of cytokine activation is the cause or the consequence of the monoclonal antibodies (6).

Interleukin 1 acts through phosphorylation of interleukin-1 receptor-associated kinases (IRAK) by its receptor. IRAK-4 belongs to the group of serine/threonine kinases and is a crucial element of both interleukin-1 receptor (IL-1R) and Toll-like receptor (TLR) pathways in innate immune responses (7). When these receptors are activated, cytosolic adaptors, like the protein MyD88, are recruited, which in turn leads to the phosphorylation of cytosolic kinases, like IRAK-1 and -4. By this, mitogen-activated protein kinases (MAPK) and NF-κB essential modulator (NEMO) are activated. Consecutively, proinflammatory cytokines and chemokines, e.g., IL-1β, -6, -8, and -12 and tumor necrosis factor alpha (TNF-α), are synthesized (8). An inborn deficiency of IRAK-4 is known to lead to frequent invasive bacterial infections (7).

In contrast to SchS, *Castleman disease* is a rare hematological disease, characterized by excessive IL-6 production and enlarged lymph nodes. Depending on the number of affected lymph nodes, unicentric and multicentric Castleman disease (UCD and MCD) can be distinguished. Human herpesvirus 8 (HHV-8) has been associated with Castleman disease, eliciting MCD especially in immunocompromised individuals, such as HIV-positive patients. If such an origin cannot be detected, MCD is classified as idiopathic multicentric Castleman disease (iMCD) (9). Pathophysiologically, plasmablasts expressing interleukin 6 (IL-6) and other proinflammatory cytokines ultimately cause multiple-organ dysfunction. Signs include lymphadenopathy, fever, weight loss, night sweats, ascites, hepatosplenomegaly, and pleural effusions (9). Moreover, anemia and abnormal platelet counts (thrombocytosis, sometimes thrombocytopenia) occur frequently (9). UCD can be cured by surgical lymph node excision while MCD requires systemic treatment. Currently approved drugs are the anti-interleukin-6 treatments siltuximab and tocilizumab (9).

Clinically, there is also a certain resemblance to other autoinflammatory conditions, like the cryopyrin-associated periodic syndrome (CAPS), caused by a gain-of-function mutation in the NLRP3 gene (part of the inflammasome) leading to increased production of IL-1β (5). SchS and CAPS share fever, urticaria-like rash, AA amyloidosis (as major complication), and effective treatment by IL-1 blockade with anakinra, canakinumab, or rilonacept. In contrast to SchS, CAPS is an autosomal dominant disorder with onset in early childhood (5). Another clinical differential diagnosis is Sweet syndrome, which, in contrast to SchS, rather shows erythematous nodules or plaques than urticarial rash, affects predominantly middle-aged women, is often malignancy-associated or drug-induced, and usually responds well to corticosteroids (10).

As SchS and CD may present with similar signs, their differential diagnosis may be difficult in some atypical cases. The abundance of either IL-1 or IL-6 signaling may aid the diagnosis (9). Here, we describe a case where we retained ultimately the diagnosis of SchS after an initial misdiagnosis of MCD by analyzing active intracellular signal proteins of the IL-1 and the IL-6 pathway.

## Case Description

A 62-year-old man presented at our immunology outpatient clinic with recurrent urticarial rash of the whole body and fever episodes, accompanied by swelling of multiple lymph nodes, abdominal pain, and malaise. He also reported intermittent painful joints, some during strain (thumb and knee), others in rest (finger joints, shoulder, and hip). These signs had been present for the last 17 years.

Fifteen years ago, he had been diagnosed with multicentric Castleman disease (MCD), based on histology of two lymph node biopsies, showing abundance of polyclonal plasma cells and CD4+ T lymphocyte cell aggregates. Biopsies of the iliac crest, performed in 2002, 2007, and 2016, displayed a slight hypercellularity, unspecific reactive changes, a minor increase in polyclonal plasma cells, and no neoplastic infiltrate. Allegedly, an HHV-8 serology had been positive at the time of diagnosis, but PCR was twice negative in 2015 and once in 2018. Over the following years, he had received several therapies, including rituximab, thalidomide, and the anti-IL-6 antibody siltuximab, which, however, had no effect on his condition. At the time of his visit at our outpatient clinic, he was on long-term glucocorticoid therapy of 15 mg prednisolone daily, which led to a slight control of symptoms.

Additionally, our patient had a preexisting Parkinson syndrome, tinnitus, gustatory and olfactory deficiencies, and corticosteroid-associated osteoporosis. In the past, he had endured an osteosynthesis after a femoral neck fracture and an aspergillosis of the lung and maxillary sinus.

The patient was suffering from fever and rash episodes impairing his daily activities and was frustrated by the long diagnostic process and the lack of improvement by the administered therapies.

The physical examination revealed a cachectic patient with muscular atrophy, no palpable hepatosplenomegaly, and a pale rash at his trunk. Photographs made by the patient before the onset of corticosteroid therapy showed a sharply defined urticaria-like exanthema across his trunk and both upper arms. There were no signs of synovitis on joint examination; his carpometacarpal joints of the thumbs were painful on pressure.

Laboratory diagnostics showed a leukocytosis with neutrophilia (16.6 g/l), a slight anemia with hemoglobin of 11.6 g/dl, normal platelet counts, and distinct elevations of CRP (87.5 mg/l) and ESR (45 mm/h). In the serum electrophoresis, two M bands were seen and the immunofixation revealed two distinct monoclonal bands of IgM/kappa. Cryoglobulins were negative.

The local dermatopathologist was asked to review a skin biopsy made 10 years ago. He confirmed an urticaria-like reaction with the histological picture of neutrophilic urticaria.

The flow cytometry revealed a significant reduction of B cells (22 μl). Serum amyloid A and IgM were increased (446 mg/l and 10.2 g/dl, respectively). Complement C4 (0.084 g/l) and the complement activity CH50 (17 U/ml) were decreased. Moreover, the interleukin 1 receptor antagonist levels were highly elevated (755 pg/ml). See also Table 1 for reference values. Cytokine measurements (IL-1 beta, IL-2, IL-7, IL-8, IL-17, IFN-α, and TNF-α) did not show any alterations.

**TABLE 1:** Relevant laboratory results at baseline and follow-up.

	Baseline	Follow up (after 6 months)	Reference values
Thrombocytes	294*10^∧^9	190*10^∧^9	[140–440*10^∧^9/L]
Leucocytes	↑19.5*10^∧^9	8.87*10^∧^9	[4.4–11.3/L*10^∧^9/L]
Lymphocytes	↓9	↓15	[20–40%]
B cells (CD19+)	↓22	↓8	[61–415 μL]
IL-1 receptor antagonist	↑755	↑284295	[<238 pg/mL]
CRP	↑87.5	2.3	[0–5 mg/dL]
Serum amyloid A	↑446	2.8	[0–6.4 mg/L]
IgG	↓6.10	↓6.56	[7.0–16.0 g/L]
IgM	↑9.85	↑8.43	[0.4–2.3 g/L]
Kappa SFLC	↑49.10	↔	[3.3–19.4 mg/L]
M gradient 1	4.8% = 0.4 g/dl	3.3% = 0.2 g/dl	
M gradient 2	1.7% = 0.1 g/dl	2.0% = 0.2 g/dl	–
Complement C3c	0.957	↓0.727	[0.900–1.800 g/L]
Complement C4	↓0.084	↓0.079	[0.100–0.400 g/L]
Complement activity CH50	↓17.0	42.0	[31.6–57.6 U/ml]

*CRP, C-reactive protein; IgG/IgM, immunoglobulin G/M; IL-1, interleukin 1; kappa SFLC, kappa serum-free light chain.*

The synopsis of his leading signs (chronic urticarial rash, recurrent fever), the neutrophilic dermal infiltration on skin biopsy, and the laboratory results, especially the monoclonal IgM, leukocytosis, and elevated CRP, led to the suspicion of SchS. He also fulfilled the Strasbourg criteria with two out of two major and three out of four minor criteria (2).

**FIGURE 1:**
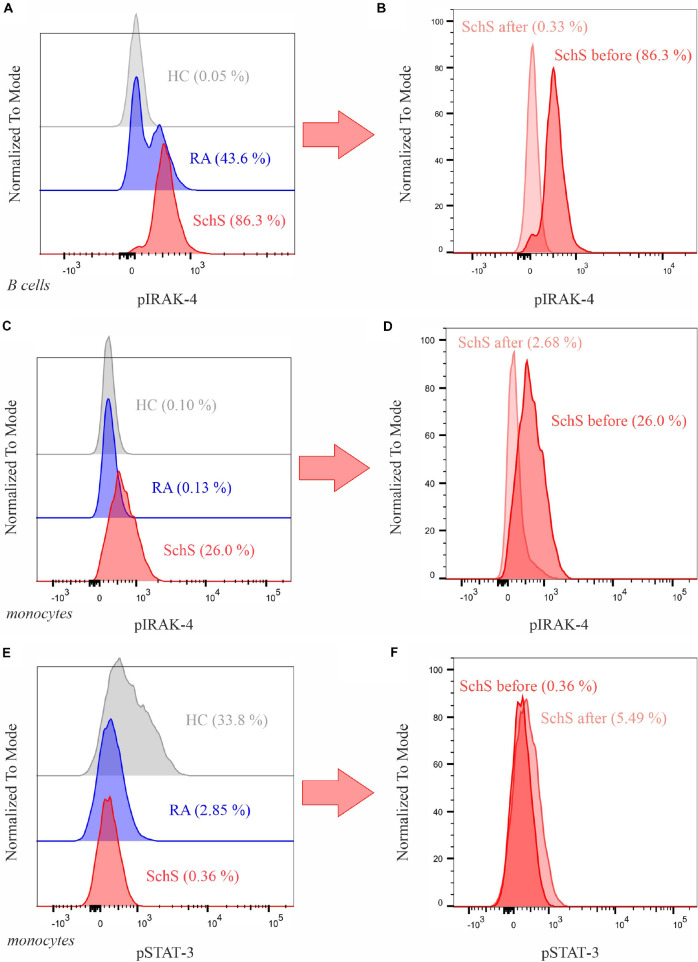
Analysis of the phosphorylation of IRAK-4 and STAT-3 in B cells and Monocytes. **(A,B)** IRAK-4 in B cells. **(C,D)** IRAK-4 in monocytes. **(E,F)** STAT-3 in monocytes. On the left, comparison of phosphorylation levels in an untreated Schnitzler syndrome patient (SchS, male, 62 years old), rheumatoid arthritis patient (RA, male, 73 years old), and healthy control (HC, male, 62 years old, with methotrexate therapy) with percentage of cells with phosphorylated target molecules in brackets; on the right, comparison of phosphorylation levels of the SchS patient before and 6 months after initiation of anakinra. Further results of healthy controls and RA patients can be seen in the Supplementary Material.

To differentiate pathology induced by IL-6 or IL-1, we tested the phosphorylation of key signaling proteins of different inflammatory pathways—STAT1–6, AKT, IRAK-4, ERK 1/2, SAPK, SMAD2/3, p38—in peripheral leukocytes using flow cytometry. Briefly, whole-blood cells were analyzed using a standardized staining protocol (BD^TM^ Phosflow Protocol for Human Whole Blood Samples, BD Biosciences; see Supplementary Material for further details). The frequency of cells with phosphorylated signaling proteins in different leukocyte populations was then determined. We found a distinct phosphorylation of IRAK-4 in 26% of monocytes (Figure 1C) and 86% of B cells (Figure 1A) but no increase of STAT-3 (Figure 1E).

Having diagnosed SchS, the patient was administered 100 mg of anakinra subcutaneously once per day while prednisolone treatment was tapered from that day on. Two months later, the patient returned to report a drastic improvement of his symptoms: the urticarial rash had completely ceased, he did not experience pain in his joints anymore, and the fever episodes had stopped. After 6 months, inflammatory markers and the extent of the monoclonal gammopathy had decreased (Table 1). Moreover, pIRAK-4 in B cells and in monocytes was reduced to a nearly normal level (Figures 1B,D), whereas STAT-3 in monocytes increased slightly from initially 0.36 to 5.49% (Figure 1F). 1.5 years after diagnosis of SchS, the patient is still on anakinra therapy without any signs of SchS.

## Discussion

Here we describe a case of a patient with SchS, initially misdiagnosed as MCD, responding to anakinra. We report that highly phosphorylated IRAK-4 may be a potential biomarker for response to IL-1 targeted treatment.

Different autoinflammatory hematological diseases may present with similar signs. Soudet et al., presented a case of a female patient with co-occurrence of MCD and SchS (11). As their patient showed recurrent fever, urticarial rash, iliac bone pain, elevated CRP, monoclonal IgM, and generalized lymphadenopathy with polytypic plasmacytosis, the diagnostic criteria principally for both diseases were fulfilled in this patient. Treatment with anakinra led to a distinct improvement of the patient’s condition (11).

In our case, we were initially not entirely certain if we encountered an overlap of the two diseases or whether the patient was simply misclassified with MCD, bearing in mind that the SchS classification criteria were definitely fulfilled. Regarding the initial lymph node biopsies, displaying an infiltration with large numbers of polyclonal plasma cells, and the patient’s other signs, the criteria for MCD were met in principal, according to the evidence-based consensus criteria by Fajgenbaum et al. (12). Nevertheless, it needs to be considered that these histologic changes are unspecific and may occur in other malignant, infectious, and autoimmune conditions, as well (12). Therefore, MCD should be rather regarded as an exclusion diagnosis. This led us to the conclusion that our case—in contrast to Soudet et al. (11)—is not another co-occurrence of these two extremely rare diseases [300 known cases worldwide of SchS and CD with an incidence of 21–25 per million patient years (13)]. In our case, cellular IL-1 signaling via pIRAK-4 exceeded IL-6 signal via pSTAT-3 supporting this evaluation. This is in line with the rapid response to anakinra but not siltuximab.

The remarkable effectiveness of IL-1 blockade is intriguing compared to the ineffective blockade of other proinflammatory cytokines, like IL-6, for which our patient received prior treatment. Blocking IL-1 induced a significant reduction of signs, which emphasizes the explicit role of the IL-1R pathway in the pathogenesis of SchS. We were able to detect phosphorylated IRAK-4, as a crucial element of this signal cascade, in a majority of B cells and monocytes in comparison to healthy controls. These results are of clinical importance as a laboratory marker that potentially distinguishes patients with SchS from patients without IL-1 overactivation has not been published so far. The fact that phosphorylation of IRAK-4 decreased after treatment also indicates its potential as a marker to measure treatment response. Although this became evident in our case, it should also be considered that IRAK-4 is not only a downstream protein of the IL-1 receptor, but also of IL-18R, IL-33R, and TLR signaling (14). Therefore, activation of these receptors may also increase pIRAK-4 levels. The increase of the IL-1R antagonist and the response to anakinra in our patient led us to the assumption of IL-1 being the major signaling pathway in this case.

In the polyclonal B-cell lymphoproliferative disorder of MCD, IL-6 rather than IL-1 signaling is implicated in the pathophysiology (15). This includes human IL-6 as well as viral IL-6, which is highly secreted especially in HHV-8-associated MCD and induces flares of the disease (16). IL-6 works via JAK/STAT signaling cascades and leads to responses of both the innate and acquired immune system in the majority of infections and inflammatory diseases (17). Notably, IL-1 functions as stimulator of IL-6 synthesis (17), which might explain why some MCD patients also hold high IL-1 levels and respond to IL-1 blockade even better than to IL-6 antagonists (9). Further research on the precise interactions of these pathways in the mentioned diseases is needed to accurately understand the molecular background of clinical overlaps as well as the reason for different treatment responses in patients with presumably the same syndromes.

We found phosphorylated IRAK-4 in 86% of our patient’s B cells, suggesting a major impact of excessive IL-1 signaling in these cells. Interestingly, the monoclonal gammopathy in our patient slightly decreased after anakinra therapy. This could imply that IL-1 stimulates the synthesis of gamma globulin in monoclonal B cells, which has been hypothesized previously (18). Activation of the IL-1 pathway resulting in the production of monoclonal immunoglobulins occurs in Waldenström’s disease, a typical lymphoproliferative complication of SchS affecting about 15% of SchS patients (2). Waldenström’s disease is an IgM-secreting lymphoplasmacytic lymphoma, which is triggered by mutation-induced overactivation of proteins of the IL-1 pathway, such as MyD88 (18).

We propose that the analysis of intracellular signaling could help to diagnose diseases with exuberant cytokine release, such as SchS, and could additionally serve as a prognostic marker for treatment response. Moreover, phosphorylated IRAK-4 was relatively increased in B cells of a patient with rheumatoid arthritis (RA) compared to a healthy control (43% and 0%, respectively). High IL-1 levels have been associated with RA (19), and anakinra is approved for this disease. Whether pIRAK-4 may also serve as a marker for anakinra responsiveness in RA patients remains to be defined. Other phosphorylated signaling molecules, such as JAK2 and 3, STAT1, 3, and 6 in different leukocyte subsets, have been examined as markers of treatment response in smaller RA collectives by (20–22). However, trials to establish IRAK-4 inhibitors as a new therapy in rheumatoid arthritis are underway (23), which underlines the possible value of this target. Analysis of phosphorylated signaling proteins by flow cytometry has been reported in various conditions, like cancer and HIV infection (24), in multiple sclerosis (25) and for the immunopathological characterization of primary immunodeficiency diseases (26, 27). Further studies are needed to test the reliability of pIRAK-4 measurements in different diseases and larger patient cohorts.

## Conclusion

We describe a case of a patient with SchS, who was misdiagnosed and treated as MCD. Excessive phosphorylation of IRAK-4 in B cells and monocytes was in line with the diagnosis of SchS and excellent response to anakinra. In unclear autoinflammatory conditions, future studies may evaluate the use of pIRAK-4 as a prognostic marker for response to IL-1 blockade.

## Data Availability Statement

All datasets presented in this study are included in the article/Supplementary Material.

## Ethics Statement

Ethical review and approval was not required for the study on human participants in accordance with the local legislation and institutional requirements. The patients/participants provided their written informed consent to participate in this study. Written informed consent was obtained from the individual(s) for the publication of any potentially identifiable images or data included in this article.

## Author Contributions

MS diagnosed and treated the patient and revised the manuscript. IH compiled the data and wrote the manuscript. PB collected data and revised the manuscript. BD conducted the pIRAK4 measurements, analyzed the results, and revised the manuscript. All authors contributed to the article and approved the submitted version.

## Conflict of Interest

The authors declare that the research was conducted in the absence of any commercial or financial relationships that could be construed as a potential conflict of interest.
