# Supplementary material for: Case Report: Extensive Phosphorylation of Interleukin-1 Receptor-Associated Kinase 4 in a Patient With Schnitzler Syndrome
Source: Front Immunol. 2020 Sep 30;11:576200. doi: 10.3389/fimmu.2020.576200 (PMC7569524; doi:10.3389/fimmu.2020.576200)
Supplement: Supplementary file 1 [file Data_Sheet_1.docx]

Supplementary Material

# Methods

Cell preparation and staining protocols for the measurement of phosphorylated signalling proteins were based on the BD™ Phosflow Protocol for Human PBMCs.

Drawn blood samples were kept on ice until further processing. 200 µl of cooled whole blood was fixed with 2 mL 1x pre-warmed Lyse/Fix Buffer (BD Biosciences Becton, Dickinson and Company, Franklin Lakes, New Jersey, USA), thoroughly mixed and incubated at 37 °C for 10 minutes. Fixed cells were washed with 2 mL phosphate-buffered saline (PBS) (Gibco, Thermo Fisher Scientific, Massachusetts, USA) at 600g for 6 minutes and permeabilized by adding 1 mL of pre-chilled BD Phosflow™ Perm Buffer III. Samples were then kept on ice for 30 minutes. 3 mL of eBioscience™ Flow Cytometry Staining Buffer (Thermo Fisher Scientific) were added to the sample and washed three times as in the previous centrifugation steps. Each sample was split and stained with monoclonal antibodies against surface and intracellular markers, see supplementary tables. Mixed samples were incubated in the dark for 60 minutes at room temperature (RT). Stained samples were then washed with Staining Buffer (BD) as in the previous centrifugation protocol. Stained cells were kept at 4 °C protected from light until cytometric measurement.

Stained cells were measured using a FACSCanto II flow cytometer (BD). 200 000 cells were measured for each sample. Flow cytometric measurements were exported as fcs-files and analysed in FlowJo (Treestar). Cells were then gated according to the gating strategy into pSTAT-3- and pIRAK-4-positive CD19^+^ B cells and Tbet^-^ monocytes (supplementary figures 1 and 2).

# Supplementary Figures and Tables

## Supplementary Tables

| *Panel 2* | **Dye** | **Company** |
| --- | --- | --- |
| **pSTAT3** | AF647 (APC) | cellsignaling |
| **pSTAT4** | PE | cellsignaling |
| **pSTAT5** | AF488 (FITC) | cellsignaling |
| **CD3** | APC-eF780 (APC-Cy7) | eBio |
| **CD4** | eF506 (AmCyan) | eBio |
| **CD45RA** | PE-Cy7 | BD |
| **Tbet** | PerCP-Cy5.5 | eBio |

Supplementary Table 1. Staining panel for measurement of pSTAT-3 in monocytes.

| *Panel 3* | **Dye** | **Company** |
| --- | --- | --- |
| **pAkt** | APC | cellsignaling |
| **pIRAK4** | PE | cellsignaling |
| **p44/42** | AF488 (FITC) | cellsignaling |
| **CD3** | PerCP | BD |
| **CD19** | V450 (PacificBlue) | BD |
| **CD16** | APC-Cy7 | BioLegend |

Supplementary Table 2. Staining panel for measurement of pIRAK-4 in B cells.

| *Panel 5* | **Dye** | **Company** |
| --- | --- | --- |
| **pAkt** | APC | cellsignaling |
| **pIRAK4** | PE | cellsignaling |
| **p44/42** | AF488 (FITC) | cellsignaling |
| **CD3** | APC-eF780 (APC-Cy7) | eBio |
| **CD4** | eF506 (AmCyan) | eBio |
| **CD45RA** | PE-Cy7 | BD |
| **Tbet** | PerCP-Cy5.5 | eBio |

Supplementary Table 3. Staining panel for measurement of pIRAK-4 in monocytes.

| **Gender** | **Age** | **pIRAK-4 pos. B cells [%]** | **pIRAK-4 pos. monocytes [%]** | **pSTAT-3 pos. monocytes [%]** |
| --- | --- | --- | --- | --- |
| f | 51 | 2,64 | 0,025 | 38,9 |
| f | 48 | 2,21 | 0 | 28,4 |
| f | 44 | 0,91 | 0,015 | 17,7 |
| m | 62 | 0,05 | 0,098 | 33,8 |
| f | 38 | 3,09 | 2,51 | 39,8 |
| f | 49 | 1,29 | 0,58 | 27,4 |
| f | 53 | 4,23 | 0,092 | 26,1 |
| m | 73 | 43,6 | 0,13 | 2,86 |
| f | 76 | 24,10 | 8,4 | 6,42 |
| f | 52 | 2,42 | 2,84 | 19,9 |
| f | 65 | 0,34 | 2,63 | 25,1 |
| f | 77 | 0,73 | 0,31 | 0,81 |
| f | 47 | 13,60 | 6,67 | 36,1 |
| m | 57 | 2,82 | 7,22 | 15,8 |
| m | 62 | 86,3 | 26 | 0,36 |

Supplementary Table 4. Gender, age and percentage of pIRAK-4 positive B cells, pIRAK-4 positive monocytes and pSTAT-3 positive monocytes of healthy controls (grey), RA patients (blue) and SchS patient (red). The order from top to bottom corresponds to the order of curves in supplementary figures 3a-c.

## Supplementary Figures


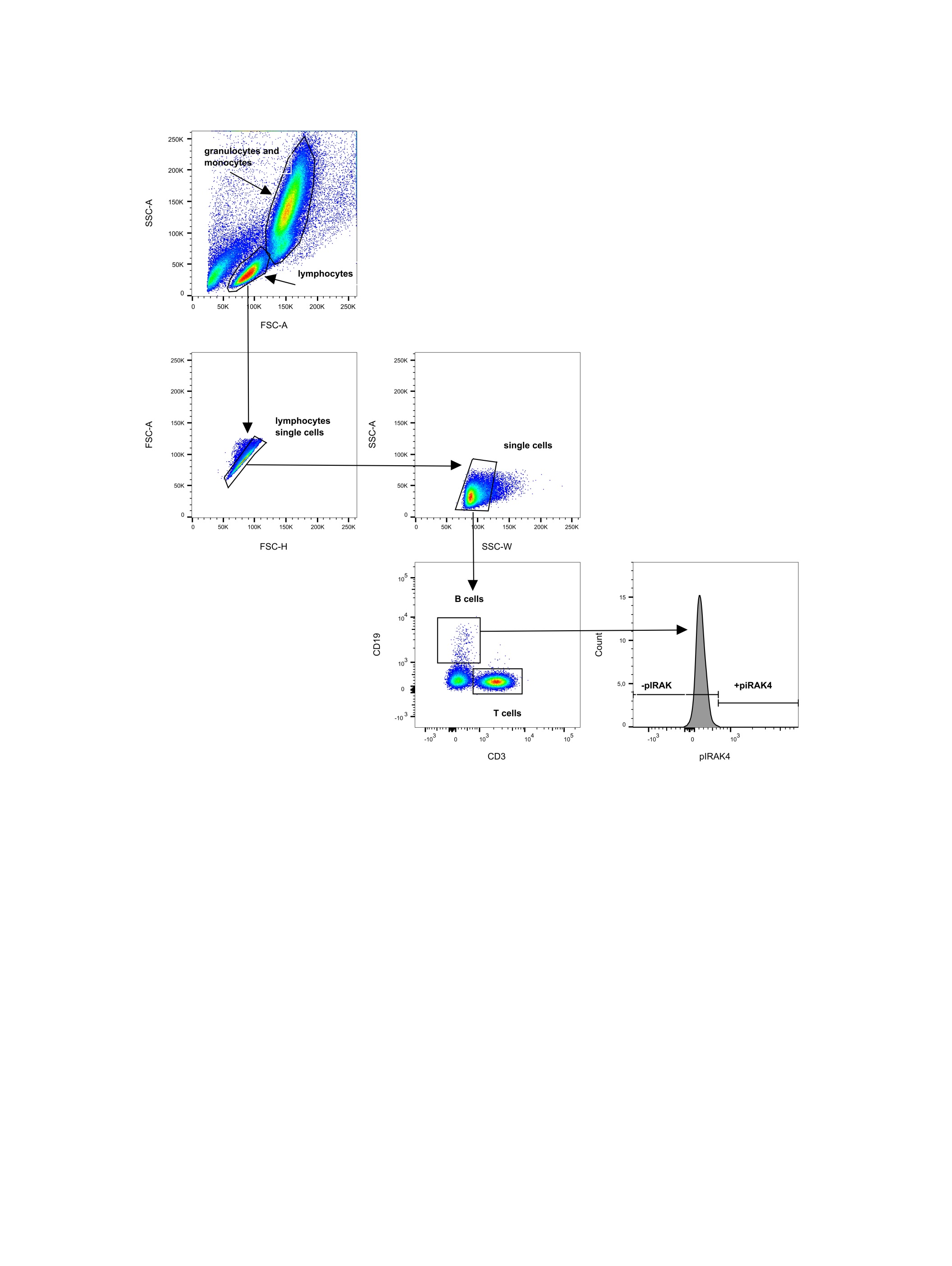


**Supplementary Figure 1.** Gating strategy to detect the percentage of pIRAK-4 positive cells in B cells. Lymphocytes were gated, B cells detected by CD19 positivity and CD3 negativity and pIRAK-4 positive cells measured in this subpopulation.


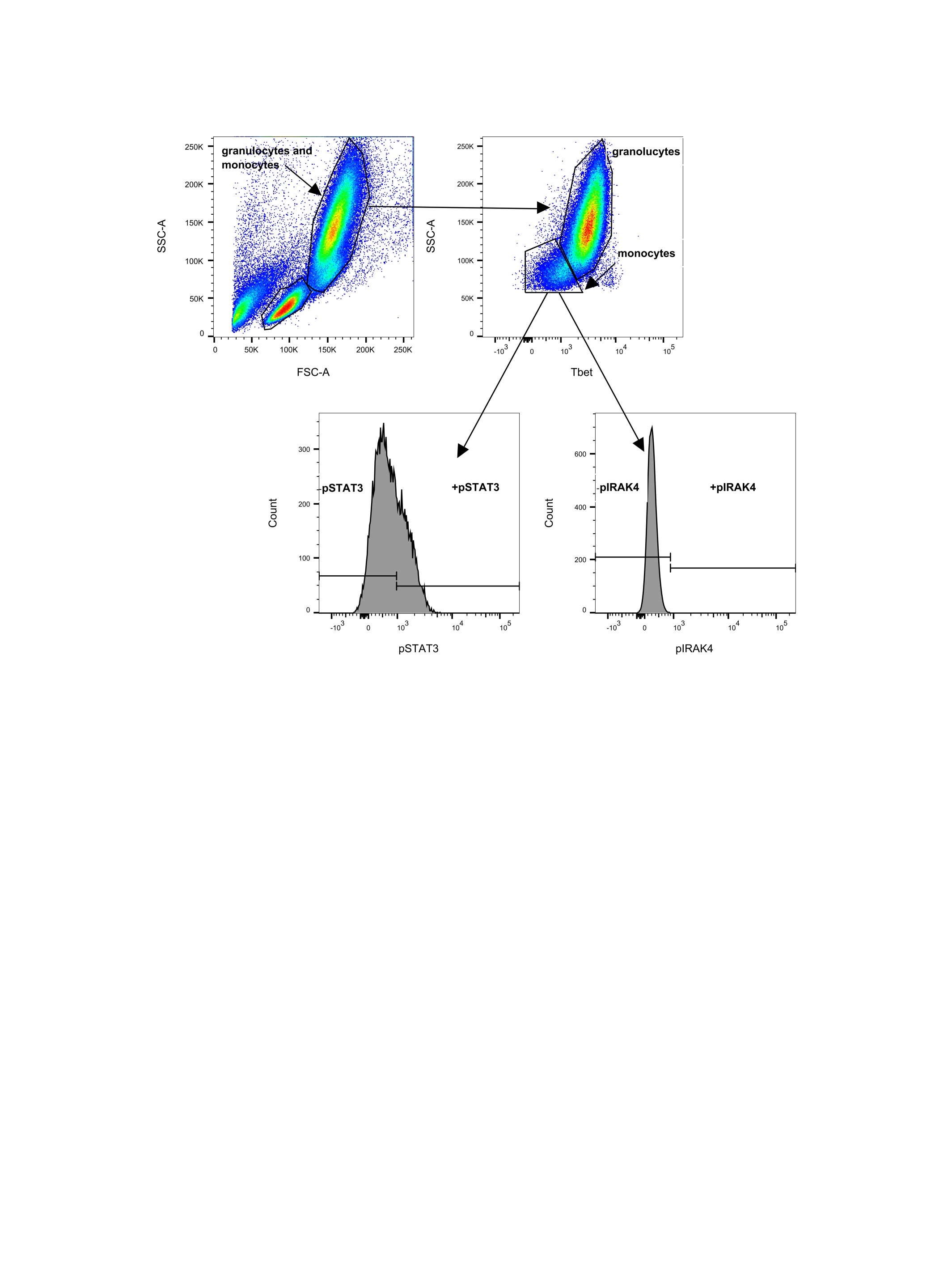


**Supplementary Figure 2.** Gating strategy to detect the percentage of pIRAK-4 positive monocytes and pSTAT-3-positive monocytes. Monocytes were gated by side and forward scatter and tbet negativity (+ CD14 positivity). Percentage of pSTAT-3 positive and pIRAK-4 positive monocytes were measured separately in two panels.

**a)
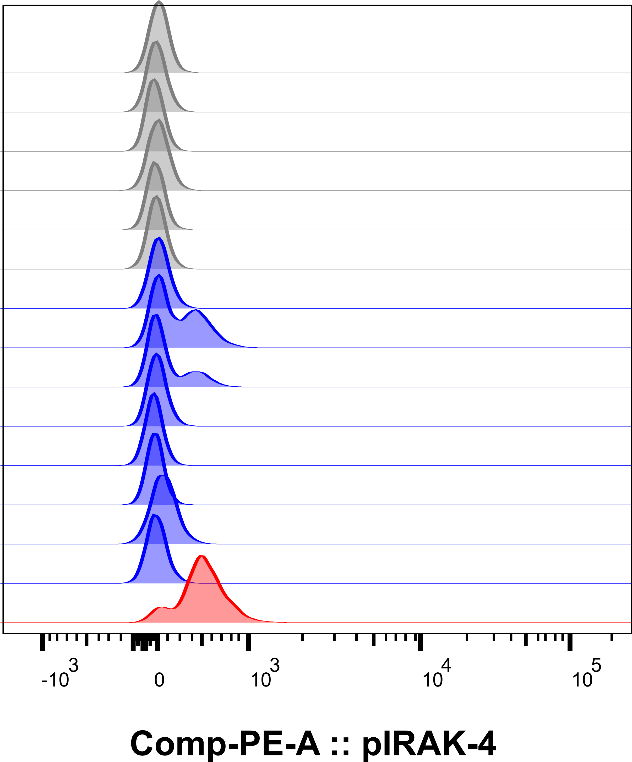
 b)
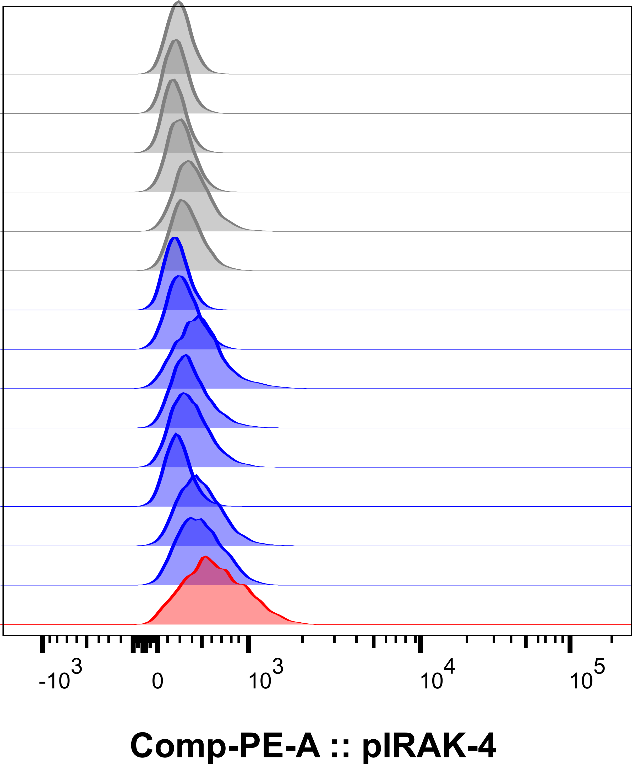
**

**c)**
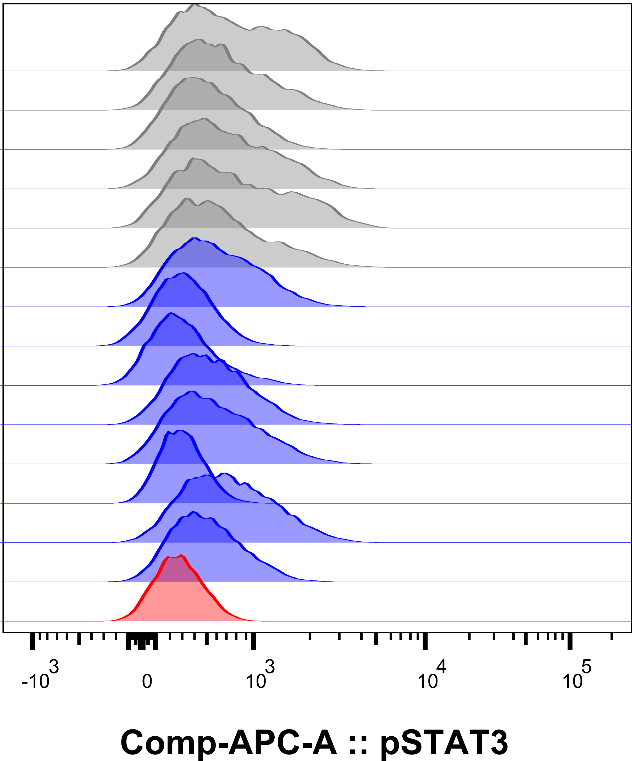


Supplementary Figure 3. Comparison of phosphorylation levels of intracellular signaling proteins between several healthy controls (grey), RA patients (blue) and SchS patient (red). The respective numbers can be seen in supplementary table 4. a) pIRAK-4 in B cells. b) pIRAK-4 in monocytes. c) pSTAT-3 in monocytes.
